# Supplementary figures and images for: Nanopore sequencing for fast determination of plasmids, phages, virulence markers, and antimicrobial resistance genes in Shiga toxin-producing Escherichia coli
Source: PLoS One. 2019 Jul 30;14(7):e0220494. doi: 10.1371/journal.pone.0220494 (PMC6667211; doi:10.1371/journal.pone.0220494)

**S1 Fig**. Annotation of plasmid 73 kb from Strain CFSAN027346 showing the antimicrobial resistance genes.


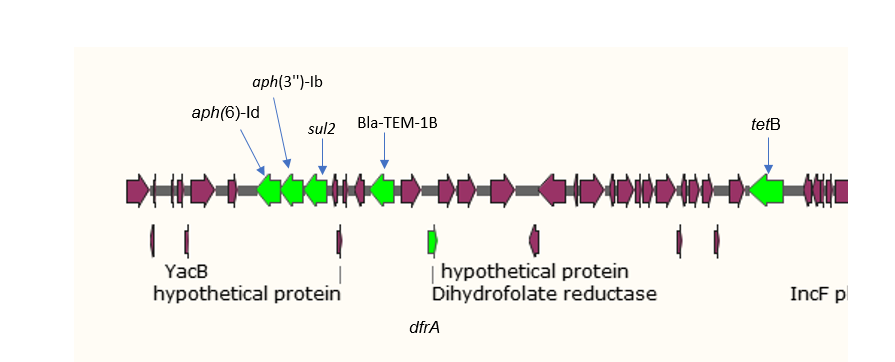

Supplement: S1 Fig — (DOCX) [file pone.0220494.s001.docx]

**S3 Fig**. Snapshot of the PHASTER (1) results for CFSAN027343 MinION chromosome.


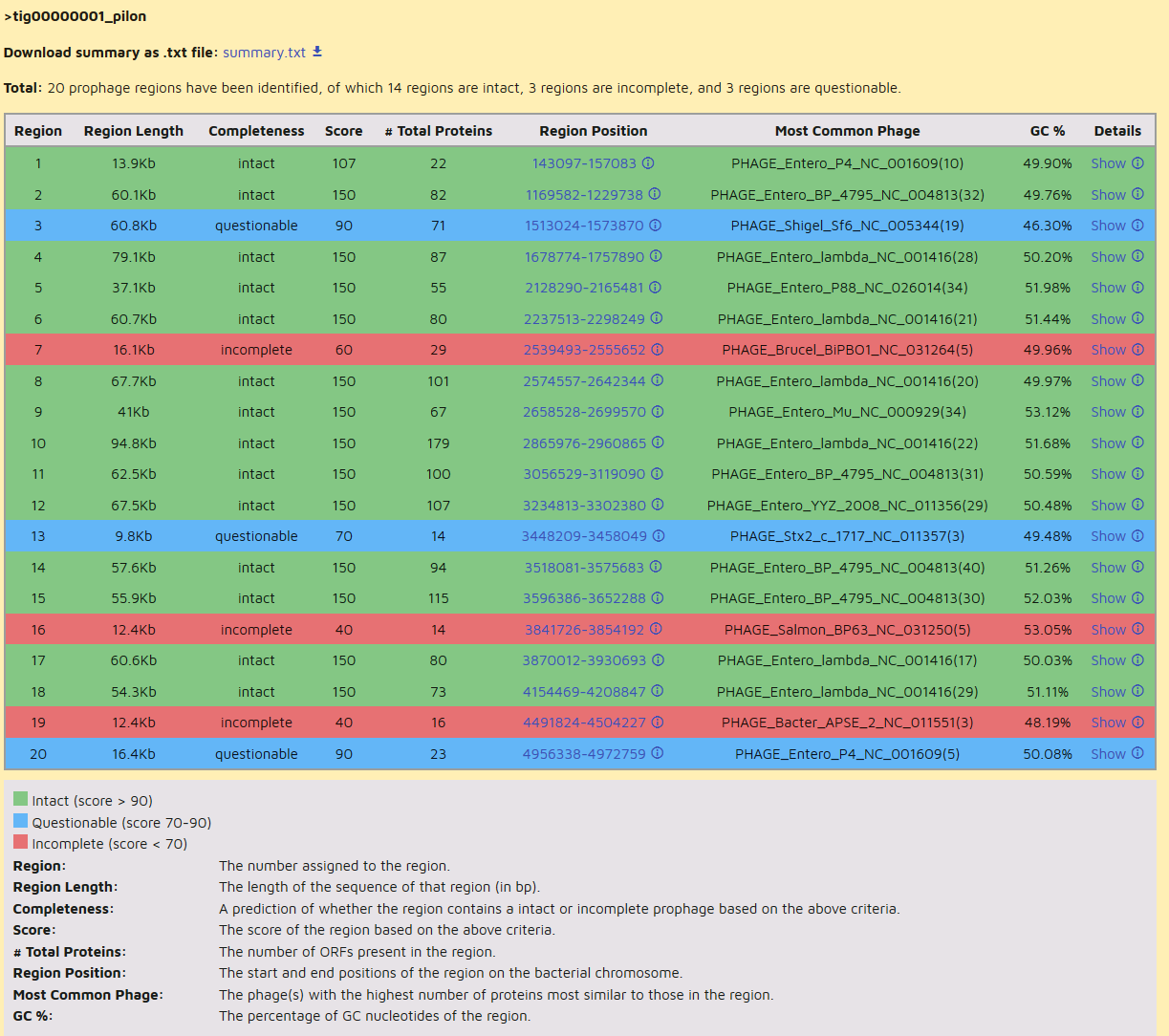

Supplement: S3 Fig — (DOCX) [file pone.0220494.s003.docx]

**S5 Fig.** Same phylogenetic tree as Fig 2A but on a larger scale to show the names of the strains.


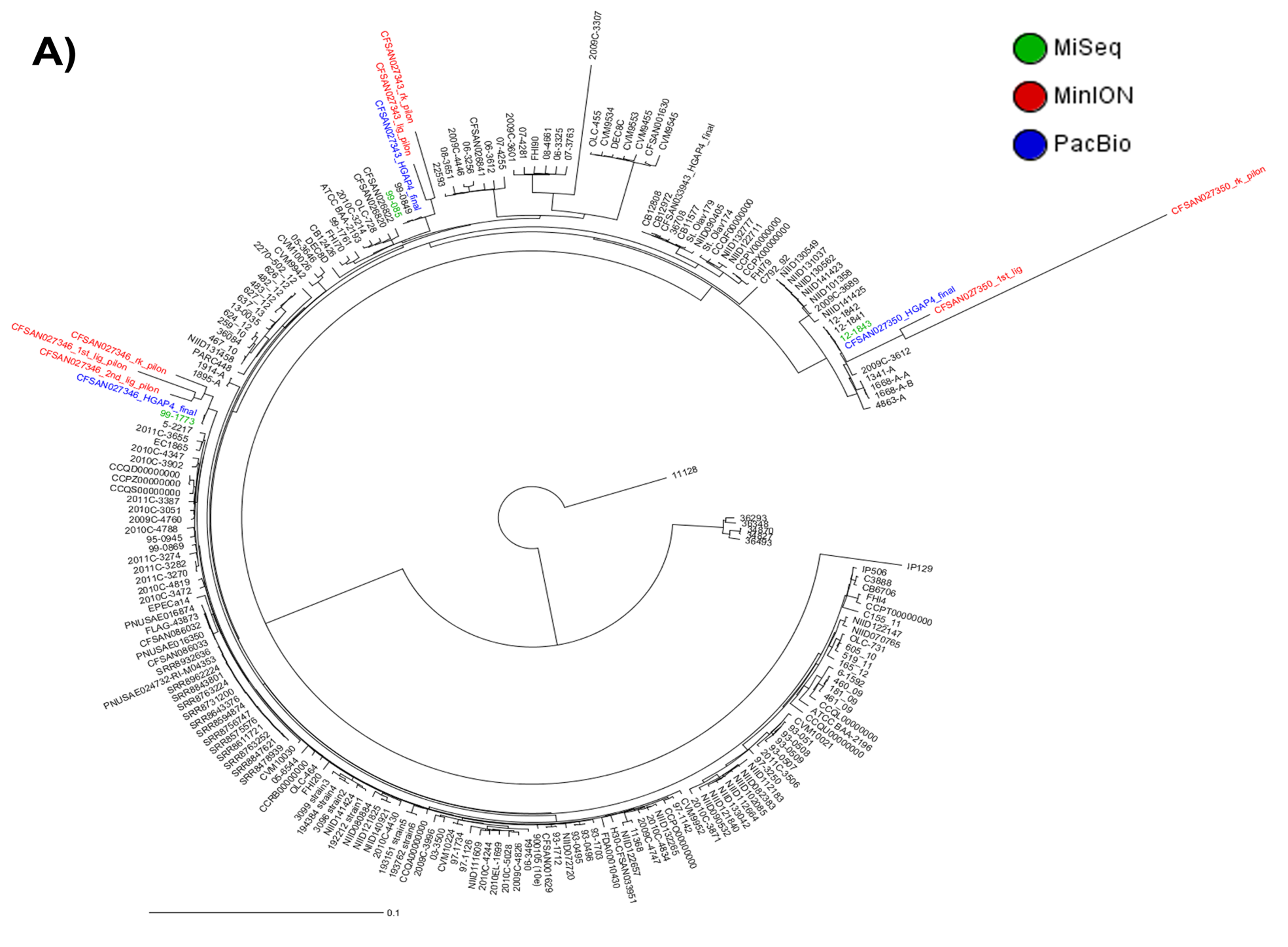

Supplement: S5 Fig — (DOCX) [file pone.0220494.s005.docx]
