# Supplementary material for: Nanopore sequencing for fast determination of plasmids, phages, virulence markers, and antimicrobial resistance genes in Shiga toxin-producing Escherichia coli
Source: PLoS One. 2019 Jul 30;14(7):e0220494. doi: 10.1371/journal.pone.0220494 (PMC6667211; doi:10.1371/journal.pone.0220494)

**S2 Fig**. SNPs differences observed by a cgMLST analysis between the genomes generated by MiSeq, PacBio, and MinION. A
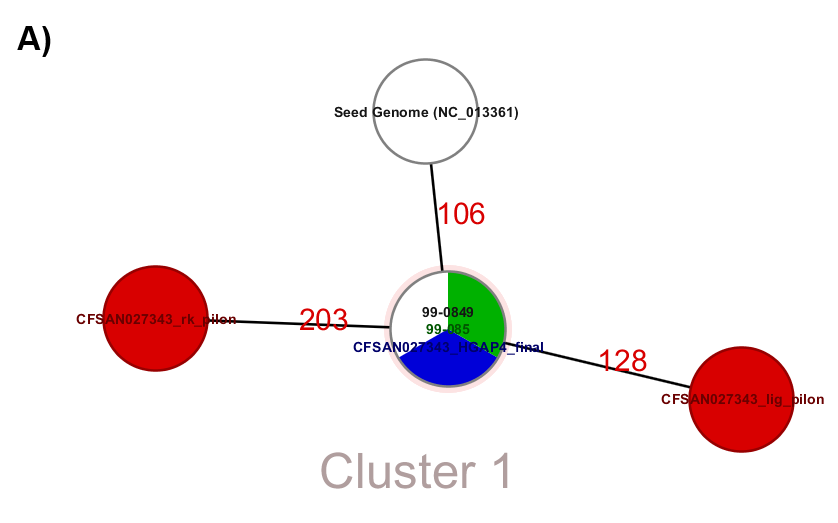
) CFSAN027343, B) CFSAN027346.


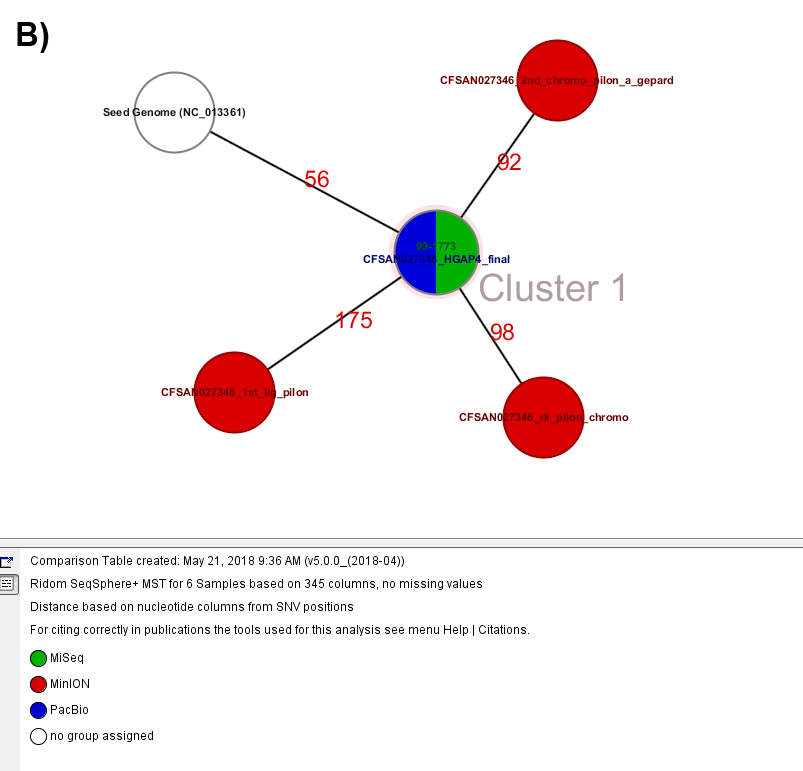

Supplement: S2 Fig — A) CFSAN027343, B) CFSAN027346. (DOCX) [file pone.0220494.s002.docx]
