# Supplementary material for: Nanopore sequencing for fast determination of plasmids, phages, virulence markers, and antimicrobial resistance genes in Shiga toxin-producing Escherichia coli
Source: PLoS One. 2019 Jul 30;14(7):e0220494. doi: 10.1371/journal.pone.0220494 (PMC6667211; doi:10.1371/journal.pone.0220494)

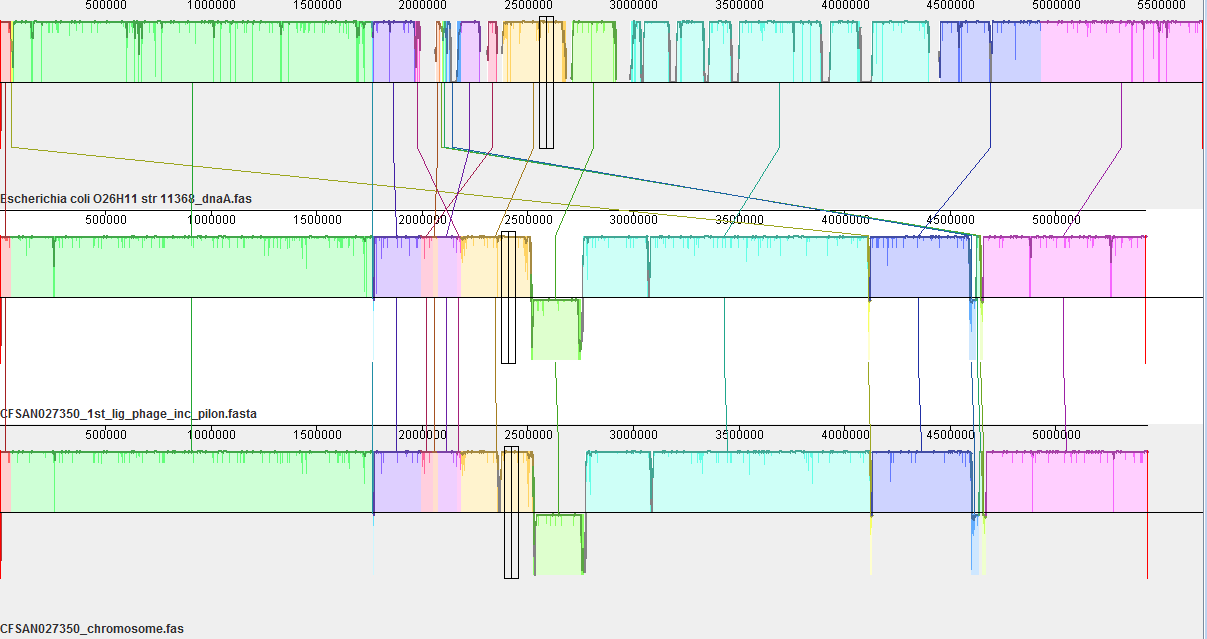
**S4 Fig.** Comparison of the synteny mapping of chromosomes generated by either MinION or PacBio, using the Sanger generated genome for STEC O26:H11 strain 11368 (AP010953) as reference with MAUVE. Each chromosome sequence is laid out in a horizontal track. Matching colors indicate homologous segments and are connected across genomes. Respective scales show the sequence coordinates in base pairs. A colored similarity plot is shown for each genome, the height of which is proportional to the level of sequence identity in that region. Strains CFSAN027346 and CFSAN027350 are shown. Top sanger sequenced strain 11368, middle MinION sequenced strain, and bottom the same strain sequenced by PacBio.


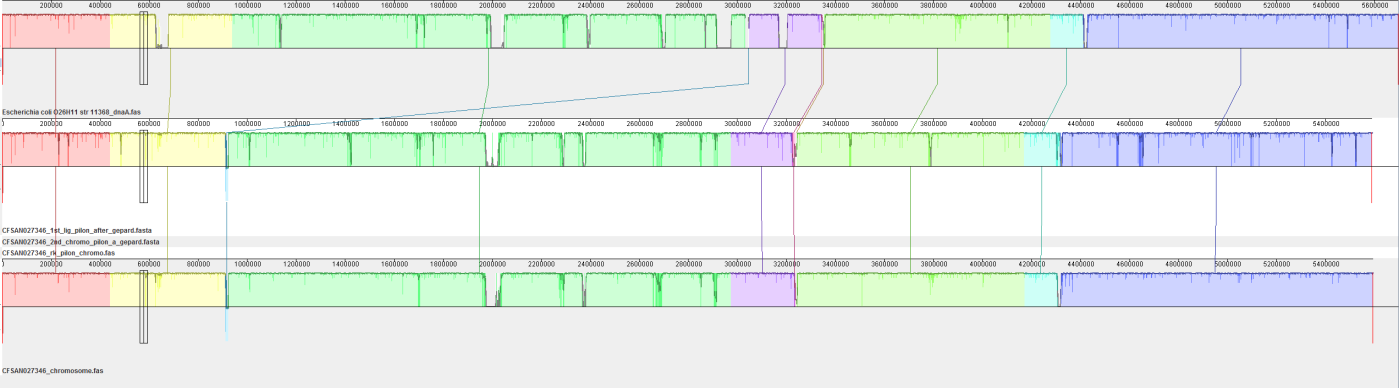

Supplement: S4 Fig — Each chromosome sequence is laid out in a horizontal track. Matching colors indicate homologous segments and are connected across genomes. Respective scales show the sequence coordinates in base pairs. A colored similarity plot is shown for each genome, the height of which is proportional to the level of sequence identity in that region. Strains CFSAN027346 and CFSAN027350 are shown. Top sanger sequenced strain 11368, middle MinION sequenced strain, and bottom the same strain sequenced by PacBio. (DOCX) [file pone.0220494.s004.docx]
