# Supplementary material for: Nanopore sequencing for fast determination of plasmids, phages, virulence markers, and antimicrobial resistance genes in Shiga toxin-producing Escherichia coli
Source: PLoS One. 2019 Jul 30;14(7):e0220494. doi: 10.1371/journal.pone.0220494 (PMC6667211; doi:10.1371/journal.pone.0220494)
Supplement: S1 Table — (DOCX) [file pone.0220494.s006.docx]

**S1 Table**. *E. coli* virulence genes tested by *in silico* virulence typing.

| **Virulence Genes** | **Description** | **Present in *E. coli* pathotype** |
| --- | --- | --- |
| *iss* | Increased serum survival | APEC, UPEC |
| *tsh* | Serine protease autotransporters of Enterobacteriaceae | APEC, UPEC |
| *nfaE* | Diffuse adherence fibrillar adhesin gene | DAEC |
| *aafA* | AAF/II major fimbrial subunit | EAEC |
| *aafB* | AAF/II minor adhesin. Enterobacteria AfaD invasin protein | EAEC |
| *aafC* | Usher, AAF/II assembly unit | EAEC |
| *aafD* | Chaperone, AAF/II assembly unit | EAEC |
| *aaiC* | secreted protein | EAEC |
| *aap* | Dispersin | EAEC |
| *aar* | AggR-activated regulator | EAEC |
| *aatA* | Dispersin transporter protein | EAEC |
| *agg3A* | AAF/III major fimbrial subunit | EAEC |
| *agg3B* | AAF/III minor adhesin. Enterobacteria AfaD invasin protein | EAEC |
| *agg3C* | Usher, AAF/III assembly unit | EAEC |
| *agg3D* | Chaperone, AAF/III assembly unit | EAEC |
| *agg4A* | AAF/IV major fimbrial subunit | EAEC |
| *agg4B* | AAF/III minor adhesin. Enterobacteria AfaD invasin protein | EAEC |
| *agg4C* | Usher, AAF/III assembly unit | EAEC |
| *agg4D* | Chaperone, AAF/III assembly unit | EAEC |
| *agg5A* | AAF/III major fimbrial subunit | EAEC |
| *aggA* | AAF/I major fimbrial subunit | EAEC |
| *aggB* | AAF/I minor adhesin. Enterobacteria AfaD invasin protein | EAEC |
| *aggC* | Usher, AAF/I assembly unit | EAEC |
| *aggD* | Chaperone, AAF/I assembly unit | EAEC |
| *aggR* | AraC transcriptional activator | EAEC |
| *air* | adhesin | EAEC |
| *astA* | Heat-stable enterotoxin 1 | EAEC, STEC, EPEC |
| *capU* | Hexosyltransferase homolog | EAEC |
| *eilA* | HilA-like regulator | EAEC |
| ORF3 | AggR-dependent genes | EAEC |
| ORF4 | AggR-dependent genes | EAEC |
| *pet* | Autotransporter enterotoxin | EAEC |
| *sepA* | Serine protease autotransporters of Enterobacteriaceae | EAEC |
| *sigA* | Serine protease autotransporters of Enterobacteriaceae | EAEC |
| *bfpA* | Major subunit of bundle-forming pili | EPEC |
| *cif* | Type III secreted effector | EPEC |
| *espC* | Serine protease autotransporters of Enterobacteriaceae | EPEC |
| *perA* | EPEC adherence factor | EPEC |
| *α-hlyA* | alpha-hemolysin in plasmid pEO5 (O26 EPEC) | EPEC |
| *lngA* | Longus type IV pilus | ETEC |
| *cfa*_*c* | Colonization factor antigen I | ETEC |
| *cofA* | Longus type IV pilus subunit | ETEC |
| *eatA* | immunogenic secreted serine protease | ETEC |
| *K88ab* | K88/F4 protein subunit | ETEC |
| *ltcA* | Heat-labile enterotoxin A subunit | ETEC |
| *sta1* | Heat-stabile enterotoxin ST-Ia | ETEC |
| *stb* | Heat-stabile enterotoxin II | ETEC |
| *ipaD* | Invasion plasmid antigen | EIEC |
| *ipaH9.8* | Type III secretion system effector ipaH7.8, E3 ubiquitin ligase | EIEC |
| *virF* | VirF transcriptional activator | EIEC |
| *prfB* | P-related fimbrial regulatory gene | Most E. coli |
| *ehxA* | Enterohemolysin | STEC |
| *espI* | Serine protease autotransporters of Enterobacteriaceae | STEC |
| *espK* | Type III secretion system | STEC |
| *espP* | Putative exoprotein precursor | STEC |
| *etpD* | Type II secretion protein | STEC |
| *iha* | Adherence protein | STEC |
| *katP* | Plasmid-encoded catalase peroxidase | STEC |
| *lpfA* | Long polar fimbriae | STEC |
| *pssA* | Protease secreted by Shiga toxin-producing E. coli (STEC) | STEC |
| *saa* | STEC autoagglutinating adhesin | STEC |
| *sab* | autotransporter of locus of enterocyte effacement-negative shiga-toxigenic | STEC |
| *stx1A* | Shiga-like toxin 1 A-subunit | STEC |
| *stx1B* | Shiga-like toxin 1 B-subunit | STEC |
| *stx2A* | Shiga toxin 2 subunit A | STEC |
| *stx2B* | Shiga toxin 2 subunit B | STEC |
| *subA* | Subtilase toxin subunit | STEC |
| *toxB* | Toxin B | STEC |
| *eae* | Intimin | STEC, EPEC |
| *efa1* | factor for adherence | STEC, EPEC |
| *espA* | Type III secretion system | STEC, EPEC |
| *espB* | Secreted protein B | STEC, EPEC |
| *espF* | Type III secretion system | STEC, EPEC |
| *espJ* | Prophage-encoded type III secretion system effector | STEC, EPEC |
| *f17A* | Subunit A of F17 fimbrial protein | STEC, EPEC |
| *f17G* | Adhesin subunit of F17 fimbriae | STEC, EPEC |
| *nleA* | Non-LEE-encoded effector A | STEC, EPEC |
| *nleB* | Non-LEE-encoded effector B | STEC, EPEC |
| *nleC* | Non-LEE-encoded effector C | STEC, EPEC |
| *tccP* | Tir cytoskeleton coupling protein | STEC, EPEC |
| *tir* | Translocated intimin receptor protein | STEC, EPEC |
| *cnf1* | Cytotoxic necrotizing factor | UPEC |
| *ireA* | Siderophore receptor | UPEC |
| *iroN* | Enterobactin siderophore receptor protein | UPEC |
| *mchB* | Microcin H47 part of colicin H | UPEC |
| *mchC* | MchC protein | UPEC |
| *mchF* | ABC transporter protein MchF | UPEC |
| *mcmA* | Microcin M part of colicin H | UPEC |
| *sat* | Serine protease autotransporters of Enterobacteriaceae | UPEC |
| *senB* | Plasmid-encoded enterotoxin | UPEC |
| *sfaS* | S-fimbrial minor subunit | UPEC |
| *vat* | vacuolating autotransporter toxin | UPEC |
| *pic* | Serine protease autotransporters of Enterobacteriaceae | UPEC, EAEC |
