# Supplementary material for: Nanopore sequencing for fast determination of plasmids, phages, virulence markers, and antimicrobial resistance genes in Shiga toxin-producing Escherichia coli
Source: PLoS One. 2019 Jul 30;14(7):e0220494. doi: 10.1371/journal.pone.0220494 (PMC6667211; doi:10.1371/journal.pone.0220494)
Supplement: S2 Table — (DOCX) [file pone.0220494.s007.docx]

**S2 Table**. Summary of ORFs in pCFSAN027350 as annotated by RAST[1].

|  | **Location** | |  |  |
| --- | --- | --- | --- | --- |
| **ORF** | **start** | **end** | **Length (bp)** | **Closest protein match** |
| 1 | 1 | 162 | 162 | RepFIB replication protein A |
| 2 | 228 | 734 | 507 | RepFIB replication protein A |
| 3 | 803 | 1453 | 651 | Mobile element protein |
| 4 | 1453 | 1800 | 348 | Mobile element protein |
| 5 | 1820 | 3391 | 1572 | Mobile element protein |
| 6 | 3447 | 3689 | 243 | RepFIB replication protein A |
| 7 | 5377 | 3839 | 1539 | Mobile element protein |
| 8 | 5774 | 5427 | 348 | Mobile element protein |
| 9 | 6172 | 5771 | 402 | Mobile element protein |
| 10 | 6571 | 6446 | 126 | hypothetical protein |
| 11 | 6680 | 6868 | 189 | Protein sok |
| 12 | 7599 | 6880 | 720 | PsiA protein |
| 13 | 8030 | 7596 | 435 | Adenine-specific methyltransferase (EC 2.1.1.72) |
| 14 | 9746 | 8085 | 1662 | FIG00638906: hypothetical protein |
| 15 | 9752 | 10168 | 417 | Phage EaA protein |
| 16 | 10319 | 10486 | 168 | Mobile element protein |
| 17 | 11329 | 10511 | 819 | Mobile element protein |
| 18 | 11667 | 11365 | 303 | Mobile element protein |
| 19 | 11776 | 12162 | 387 | Mobile element protein |
| 20 | 12354 | 12187 | 168 | Mobile element protein |
| 21 | 12460 | 12729 | 270 | Mobile element protein |
| 22 | 22529 | 13020 | 9510 | FIG00640240: hypothetical protein |
| 23 | 23208 | 22798 | 411 | Mobile element protein |
| 24 | 23274 | 23390 | 117 | hypothetical protein |
| 25 | 23360 | 23749 | 390 | Mobile element protein |
| 26 | 24233 | 24508 | 276 | Mobile element protein |
| 27 | 24505 | 25395 | 891 | Mobile element protein |
| 28 | 25975 | 25526 | 450 | Antitoxin 1 |
| 29 | 26170 | 25988 | 183 | hypothetical protein |
| 30 | 26788 | 26910 | 123 | hypothetical protein |
| 31 | 27283 | 27017 | 267 | HicB protein |
| 32 | 27987 | 27703 | 285 | FIG00644782: hypothetical protein |
| 33 | 28147 | 28320 | 174 | hypothetical protein |
| 34 | 28794 | 28531 | 264 | FIG00640806: hypothetical protein |
| 35 | 29032 | 28772 | 261 | FIG00644864: hypothetical protein |
| 36 | 29614 | 29405 | 210 | hypothetical protein |
| 37 | 29978 | 29655 | 324 | FIG00640079: hypothetical protein |
| 38 | 30900 | 30508 | 393 | Phage protein |
| 39 | 31223 | 30933 | 291 | FIG00641150: hypothetical protein |
| 40 | 31311 | 31607 | 297 | Mobile element protein |
| 41 | 31607 | 32794 | 1188 | Mobile element protein |
| 42 | 33138 | 32974 | 165 | Mobile element protein |
| 43 | 33610 | 33245 | 366 | Mobile element protein |
| 44 | 34205 | 34528 | 324 | Transposase |
| 45 | 34681 | 34544 | 138 | Mobile element protein |
| 46 | 35046 | 34837 | 210 | Mobile element protein |
| 47 | 35475 | 35143 | 333 | Mobile element protein |
| 48 | 36196 | 45141 | 8946 | RTX toxins determinant A and related Ca2+-binding proteins |
| 49 | 45305 | 45580 | 276 | Mobile element protein |
| 50 | 45577 | 46467 | 891 | Mobile element protein |
| 51 | 46782 | 46904 | 123 | hypothetical protein |
| 52 | 47208 | 47074 | 135 | hypothetical protein |
| 53 | 47185 | 47319 | 135 | regulatory protein |
| 54 | 47433 | 47657 | 225 | hypothetical protein |
| 55 | 47954 | 48745 | 792 | Polysaccharide deacetylase |
| 56 | 48745 | 49851 | 1107 | Hexosyltransferase homolog |
| 57 | 49941 | 51662 | 1722 | UPF0141 membrane protein YijP |
| 58 | 51703 | 52734 | 1032 | Lipid A biosynthesis (KDO) 2-(lauroyl)-lipid IVA acyltransferase |
| 59 | 52898 | 53272 | 375 | Mobile element protein |
| 60 | 53662 | 53276 | 387 | Mobile element protein |
| 61 | 55346 | 53775 | 1572 | Mobile element protein |
| 62 | 55713 | 55366 | 348 | Mobile element protein |
| 63 | 56363 | 55713 | 651 | Mobile element protein |
| 64 | 57601 | 56432 | 1170 | Mobile element protein |
| 65 | 57982 | 57632 | 351 | Mobile element protein |
| 66 | 58341 | 57979 | 363 | Mobile element protein |
| 67 | 58455 | 58730 | 276 | Mobile element protein |
| 68 | 58727 | 59617 | 891 | Mobile element protein |
| 69 | 59771 | 60208 | 438 | Mobile element protein |
| 70 | 60258 | 60377 | 120 | Mobile element protein |
| 71 | 61952 | 60381 | 1572 | Mobile element protein |
| 72 | 62319 | 61972 | 348 | Mobile element protein |
| 73 | 62996 | 62319 | 678 | Mobile element protein |
| 74 | 65210 | 64410 | 801 | Mobile element protein |
| 75 | 65533 | 65207 | 327 | Mobile element protein |
| 76 | 65738 | 66091 | 354 | FIG00241420: hypothetical protein |
| 77 | 66654 | 66253 | 402 | VagD |
| 78 | 66896 | 66666 | 231 | Virulence-associated protein vagC |
| 79 | 66853 | 67002 | 150 | hypothetical protein |
| 80 | 67152 | 67033 | 120 | hypothetical protein |
| 81 | 67343 | 67149 | 195 | hypothetical protein |
| 82 | 67418 | 67870 | 453 | hypothetical protein |
| 83 | 67860 | 68654 | 795 | Resolvase |
| 84 | 68799 | 69179 | 381 | FIG00643174: hypothetical protein |
| 85 | 71021 | 69582 | 1440 | Microcin H47 secretion protein |
| 86 | 73145 | 71025 | 2121 | Methionine ABC transporter ATP-binding protein |
| 87 | 76191 | 73195 | 2997 | hemolysin-type calcium-binding region |
| 88 | 76708 | 76193 | 516 | RTX toxin activating lysine-acyltransferase (EC 2.3.1.-) |
| 89 | 77157 | 77005 | 153 | Phage protein |
| 90 | 77148 | 77555 | 408 | Mobile element protein |
| 91 | 77548 | 78039 | 492 | Mobile element protein |
| 92 | 78209 | 78042 | 168 | hypothetical protein |
| 93 | 78388 | 78600 | 213 | hypothetical protein |
| 94 | 79575 | 78952 | 624 | FIG00641946: hypothetical protein |
| 95 | 79734 | 79853 | 120 | putative nuclease |
| 96 | 79850 | 80227 | 378 | putative nuclease |
| 97 | 80566 | 81240 | 675 | IncF plasmid conjugative transfer pilin acetylase TraX |
| 98 | 81530 | 82159 | 630 | Dienelactone hydrolase and related enzymes |
| 99 | 82214 | 82822 | 609 | IncF plasmid conjugative transfer fertility inhibition protein FinO |
| 100 | 82953 | 83165 | 213 | FIG00640597: hypothetical protein |
| 101 | 83349 | 83870 | 522 | FIG01068181: hypothetical protein |
| 102 | 83916 | 84125 | 210 | Haemolysin expression modulating protein paralog |
| 103 | 84163 | 84753 | 591 | YihA |
| 104 | 84993 | 85247 | 255 | Replication regulatory protein repA2 (Protein copB) |
| 105 | 85538 | 86407 | 870 | RepA1 |
| 106 | 87143 | 87265 | 123 | FIG00643981: hypothetical protein |
| 107 | 87320 | 87589 | 270 | Prevent host death protein, Phd antitoxin # D |
| 108 | 87586 | 87867 | 282 | YacB |
| 109 | 88370 | 88254 | 117 | hypothetical protein |
| 110 | 89283 | 88381 | 903 | Mobile element protein |
| 111 | 89842 | 89570 | 273 | FIG01069941: hypothetical protein |
| 112 | 89836 | 89979 | 144 | hypothetical protein |
| 113 | 90083 | 89967 | 117 | hypothetical protein |
| 114 | 90996 | 90094 | 903 | Mobile element protein |
| 115 | 91555 | 91283 | 273 | FIG01069941: hypothetical protein |
| 116 | 91655 | 91777 | 123 | hypothetical protein |
| 117 | 91908 | 92717 | 810 | Colanic acid biosynthesis acetyltransferase WcaB (EC 2.3.1.-) |
| 118 | 94341 | 93154 | 1188 | Mobile element protein |
| 119 | 94637 | 94341 | 297 | Mobile element protein |
| 120 | 94762 | 94875 | 114 | Single-stranded DNA-binding protein |
| 121 | 96169 | 94982 | 1188 | Mobile element protein |
| 122 | 96465 | 96169 | 297 | Mobile element protein |
| 123 | 96590 | 96703 | 114 | Single-stranded DNA-binding protein |
| 124 | 97997 | 96810 | 1188 | Mobile element protein |
| 125 | 98293 | 97997 | 297 | Mobile element protein |
| 126 | 98543 | 98397 | 147 | Mobile element protein |
| 127 | 98913 | 99128 | 216 | Mobile element protein |
| 128 | 99207 | 99416 | 210 | Mobile element protein |
| 129 | 99726 | 99869 | 144 | FIG01048508: hypothetical protein |
| 130 | 99876 | 100235 | 360 | FIG01048508: hypothetical protein |
| 131 | 100441 | 100253 | 189 | FIG00639560: hypothetical protein |
| 132 | 100580 | 100455 | 126 | hypothetical protein |
| 133 | 100715 | 101371 | 657 | Adenine-specific methyltransferase (EC 2.1.1.72) |
| 134 | 101372 | 101593 | 222 | putative cytoplasmic protein |
| 135 | 101607 | 102041 | 435 | YcgB |
| 136 | 102086 | 102856 | 771 | FIG00638373: hypothetical protein |
| 137 | 103279 | 102986 | 294 | hypothetical protein |
| 138 | 103274 | 103699 | 426 | Putative antirestriction protein |
| 139 | 103746 | 104168 | 423 | Adenine-specific methyltransferase (EC 2.1.1.72) |
| 140 | 104165 | 104356 | 192 | FIG00638431: hypothetical protein |
| 141 | 104353 | 104475 | 123 | hypothetical protein |
| 142 | 104879 | 104631 | 249 | FIG00244619: hypothetical protein |
| 143 | 105032 | 105172 | 141 | Single-stranded DNA-binding protein |
| 144 | 105159 | 105461 | 303 | Single-stranded DNA-binding protein |
| 145 | 106755 | 105568 | 1188 | Mobile element protein |
| 146 | 107051 | 106755 | 297 | Mobile element protein |
| 147 | 107176 | 107289 | 114 | Single-stranded DNA-binding protein |
| 148 | 108583 | 107396 | 1188 | Mobile element protein |
| 149 | 108879 | 108583 | 297 | Mobile element protein |
| 150 | 109067 | 110014 | 948 | Mobile element protein |
| 151 | 110038 | 110259 | 222 | FIG01048117: hypothetical protein |
| 152 | 110541 | 110290 | 252 | FIG00642528: hypothetical protein |
| 153 | 110772 | 110575 | 198 | FIG01046993: hypothetical protein |
| 154 | 110889 | 110776 | 114 | hypothetical protein |
| 155 | 110915 | 111079 | 165 | hypothetical protein |
| 156 | 111121 | 111453 | 333 | Z1226 protein |
| 157 | 111478 | 111969 | 492 | Z1226 protein |
| 158 | 112390 | 112055 | 336 | FIG01047424: hypothetical protein |
| 159 | 112496 | 112380 | 117 | hypothetical protein |
| 160 | 112593 | 112955 | 363 | periplasmic nickel-binding protein NikA |
| 161 | 113039 | 115678 | 2640 | involved in conjugative DNA transfer |
| 162 | 115746 | 116177 | 432 | FIG00638738: hypothetical protein |
| 163 | 116415 | 116275 | 141 | hypothetical protein |
| 164 | 116559 | 116870 | 312 | Mobile element protein |
| 165 | 116883 | 117065 | 183 | hypothetical protein |
| 166 | 117059 | 117736 | 678 | Mobile element protein |
| 167 | 118126 | 117812 | 315 | hypothetical protein |
| 168 | 118119 | 118796 | 678 | Mobile element protein |
| 169 | 118796 | 119143 | 348 | Mobile element protein |
| 170 | 119163 | 120734 | 1572 | Mobile element protein |
| 171 | 123228 | 120925 | 2304 | FIG00640314: hypothetical protein |
| 172 | 124327 | 123209 | 1119 | TrbB protein |
| 173 | 125559 | 124324 | 1236 | TrbA |
| 174 | 125651 | 125833 | 183 | hypothetical protein |
| 175 | 125988 | 126128 | 141 | hypothetical protein |
| 176 | 126650 | 126378 | 273 | Neurotensin receptor R8 |
| 177 | 126908 | 126717 | 192 | FIG00643543: hypothetical protein |
| 178 | 127283 | 126921 | 363 | FIG00641173: hypothetical protein |
| 179 | 127485 | 127294 | 192 | hypothetical protein |
| 180 | 127617 | 128294 | 678 | Mobile element protein |
| 181 | 128294 | 128641 | 348 | Mobile element protein |
| 182 | 128661 | 130232 | 1572 | Mobile element protein |
| 183 | 131896 | 130358 | 1539 | Mobile element protein |
| 184 | 132293 | 131946 | 348 | Mobile element protein |
| 185 | 132691 | 132290 | 402 | Mobile element protein |
| 186 | 132889 | 132746 | 144 | hypothetical protein |
| 187 | 136641 | 132946 | 3696 | Per-activated serine protease autotransporter enterotoxin EspC |
| 188 | 137728 | 137225 | 504 | putative nuclease |
| 189 | 137888 | 138511 | 624 | FIG00641946: hypothetical protein |
| 190 | 138599 | 138976 | 378 | hypothetical protein |
| 191 | 139368 | 138973 | 396 | Mobile element protein |
| 192 | 139517 | 139395 | 123 | Mobile element protein |
| 193 | 140031 | 139720 | 312 | DNA-directed RNA polymerase, sigma subunit (sigma70/sigma32) |
| 194 | 140199 | 140354 | 156 | hypothetical protein |
| 195 | 140952 | 140305 | 648 | surface exclusion protein |
| 196 | 143104 | 141023 | 2082 | IncI1 plasmid conjugative transfer integral membrane protein TraY |
| 197 | 143830 | 143261 | 570 | IncI1 plasmid conjugative transfer protein TraX |
| 198 | 144945 | 143827 | 1119 | IncI1 plasmid conjugative transfer protein TraW |
| 199 | 145430 | 144993 | 438 | IncI1 plasmid conjugative transfer protein TraV |
| 200 | 145420 | 145638 | 219 | hypothetical protein |
| 201 | 147949 | 145610 | 2340 | IncI1 plasmid conjugative transfer protein TraU |
| 202 | 147938 | 148153 | 216 | hypothetical protein |
| 203 | 149702 | 148164 | 1539 | Mobile element protein |
| 204 | 150098 | 149751 | 348 | Mobile element protein |
| 205 | 150499 | 150095 | 405 | Mobile element protein |
| 206 | 151924 | 150737 | 1188 | Mobile element protein |
| 207 | 152220 | 151924 | 297 | Mobile element protein |
| 208 | 152308 | 152973 | 666 | Mobile element protein |
| 209 | 154156 | 153194 | 963 | Chromosome (plasmid) partitioning protein ParB |
| 210 | 155358 | 154153 | 1206 | Chromosome (plasmid) partitioning protein ParA |
| 211 | 155585 | 155950 | 366 | FIG00641067: hypothetical protein |
| 212 | 156074 | 156325 | 252 | RelB/StbD replicon stabilization protein (antitoxin to RelE/StbE) |
| 213 | 156322 | 156609 | 288 | RelE/StbE replicon stabilization toxin |
| 214 | 157223 | 156996 | 228 | hypothetical protein |

References

1. Aziz RK, Bartels D, Best AA, DeJongh M, Disz T, Edwards RA et al. The RAST Server: rapid annotations using subsystems technology. BMC Genomics. 2008; 9: 75. 1471-2164-9-75;10.1186/1471-2164-9-75.
